# Depression and Anxiety in Times of COVID-19: How Coping Strategies and Loneliness Relate to Mental Health Outcomes and Academic Performance

**DOI:** 10.3389/fpsyg.2021.682684

**Published:** 2021-10-25

**Authors:** Sebastian Freyhofer, Niklas Ziegler, Elisabeth M. de Jong, Michaéla C. Schippers

**Affiliations:** Department of Technology and Operations Management, Rotterdam School of Management, Erasmus University, Rotterdam, Netherlands

**Keywords:** COVID-19, depression, anxiety, loneliness, coping strategies, academic performance, procrastination

## Abstract

The link between depression, anxiety, and loneliness has been well established in the literature. Yet, the performance consequences of these negative mental health outcomes and the role of coping behaviors, as well as behavioral consequences such as procrastination as mediators have received far less research attention. Due to the COVID-19 social isolation restrictions, people are at risk of falling into a negative mental health spiral that can also affect their performance over time. The purpose of this longitudinal study among 881 first-year bachelor students is to explore the mechanisms by which loneliness, coping strategies in the context of COVID-19, mental health outcomes and procrastination sequentially mediate the relationship depression and anxiety on the one hand, and academic performance on the other hand. We measured mental health variables several times during the COVID-19 crisis and assessed how this translates into academic performance at the end of the academic year. By performing exploratory and confirmatory factor analyses, three high-order factors for the coping strategies in the context of the COVID-19 crisis were identified, namely maladaptive coping, adaptive coping, and supportive coping. Structural equation modeling was used to test the sequential mediational model. The results showed that maladaptive coping strategies employed at T2 during the lockdown, but not adaptive or supportive coping partially mediate the trajectories of depression (T1) and anxiety (T1). Loneliness (T2) partially mediated the trajectory of depression and anxiety (T1), and procrastination fully mediated the impact of depression (T3) on academic performance (T4). These results help understand the mechanisms that influence mental health and academic performance outcomes in response to the COVID-19 crisis. Based on the study outcomes, educational researchers can test strategies to reduce the adverse effects of stressful situations in learning environments by targeting maladaptive coping behaviors and procrastination.

## Introduction

Since the start of the COVID-19 pandemic, many countries have taken restrictive measures and have implemented lockdowns in an effort to contain the spread of the virus. The pandemic and these restrictions have changed living conditions across countries and age groups, affecting social life and increasing mental health issues (Kumar and Nayar, 2020), which may even have caused people to experience grief over the loss of their normal lives (de Jong et al., 2020). In addition, authors have found various adverse side effects, including mental and physical health issues (for reviews see Schippers, 2020a,b).

**FIGURE 1:**
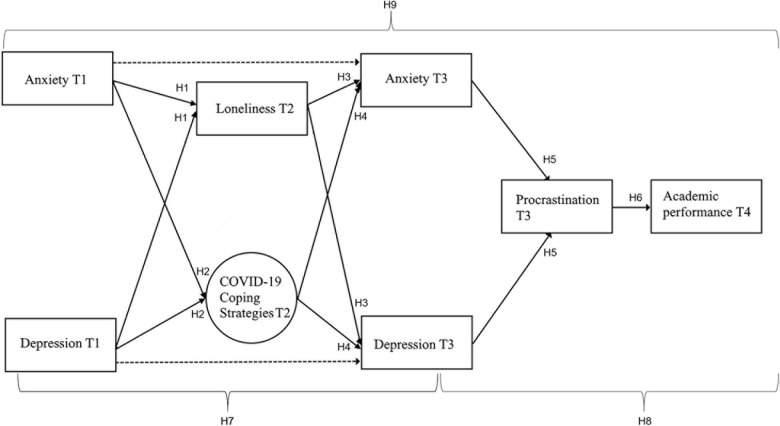
Hypothesized sequential mediation model including the three higher-order coping strategies in the context of the COVID-19 pandemic.

For young people, the crisis and accompanying measures often have negative effects on their psychological well-being (Wang Z. H. et al., 2020). In the context of higher education, COVID-19 has affected more than 89% of higher education institutions in the world (IAU, 2020). Most of these institutions had to switch to online mode practically overnight, leading to a severe reduction in or even the elimination of in-person interactions. Furthermore, the pandemic has seriously impacted student’s mental health and continues to do so (Tang et al., 2020; Wang C. et al., 2020; Fruehwirth et al., 2021). Before the pandemic started, research had shown that during the transition to higher education, students were already at risk of developing mental health issues (Hunt and Eisenberg, 2010; Auerbach et al., 2018), which in turn increased the risk of procrastination and academic underperformance (Bruffaerts et al., 2018). Although there is some consensus in the literature on the impacts of the pandemic on student’s mental health, there are equivocal findings with regard to the question whether these impacts prevail after the lockdowns are released. One study found that easing restrictions does not have a significant impact on improving mental health (Pieh et al., 2021), e.g., while others found that mental health returns to baseline after releasing the pandemic restrictions (Meda et al., 2021). A large longitudinal study among 157,213 Americans (Yarrington et al., 2021) tracked the mental health outcomes during 5 months, from before the stay-at-home orders, up to two and a half months (on average) after the ease of the restrictions. They found a difference on the trajectories of anxiety and depression; where anxiety levels decreased, surprisingly, depression continued increasing after relaxing the stay-at-home orders. Although coping strategies were not measured, the authors theorized that coping may play an important role protecting against negative long-term mental health outcomes, as levels of anxiety returned to baseline after stay-at-home measures were relaxed, they speculated that this was caused by effective coping strategies. Thus, there is a strong need for more knowledge on how students cope with the situation, and how their coping relates to their psychological well-being and academic performance. The most important aims of this paper are therefore (a) to examine the effect of the COVID-19 crisis on psychological well-being and academic performance of students in higher education over time, (b) to examine the role of coping strategies and loneliness on psychological well-being, and (c) to assess the mediating role of procrastination between psychological well-being and academic performance. Furthermore, we examined the factor structure of coping strategies in the context of the COVID-19 crisis (see Figure 1). As coping strategies have been found to vary in their usefulness and how they associate with one another depending on the context (Krägeloh, 2011), this is to the best of our knowledge the first study to assess how the pandemic has influenced their aggregation in this new context and their integration into a more comprehensive model including mental health and academic performance outcomes. Greater insights into the different coping strategies that students apply in the context of the COVID-19 crisis and the relation between those coping strategies and students’ mental health could help mental-health care professionals to offer a more tailored support to students.

## Theoretical Framework

The COVID-19 crisis has become a source of stress, which includes threats to human life and social disruptions, as the population has been forced to change their habits and patterns of behavior (Schippers, 2020b; Zhang et al., 2020). Although in terms of scale, this event is unprecedented, we know that these types of disastrous events are known to impact mental health, resulting in higher levels of depression (Norris et al., 2002). A recent meta-analysis by Bueno-Notivol et al. (2021) estimated that compared to the prevalence in 2017, the current crisis has produced an increase up to seven times in the levels of depression in the general population, as compared to the period before the lockdowns started. Ettman et al. (2020) came to a similar conclusion, reporting a threefold increase of depressive symptoms among the adult United States population. Furthermore, the changes to normal life have become a source of anxiety due to mobility restrictions, reduced social connections, fear for access to supplies, loss of family incomes, communication of conspiracy theories, and fear for individuals’ health conditions and that of their families (Jakovljevic et al., 2020).

Additionally, COVID-19 increases the fear of contagion from social interactions, withdrawal from routine and loss of normalcy, as well as limited interactions even within closer social circles (Brooks et al., 2020). Prior research has shown that these types of disruptions are related to heightened loneliness, depression, and anxiety (Leigh-Hunt et al., 2017). Physical mobility restrictions like quarantining and social distancing are likely to be the drivers in increasing the perception of loneliness (Wilder-Smith and Freedman, 2020), as they may increase the discrepancy between desired and perceived social relationships (Perlman and Peplau, 1981). Additionally, the impact on social interactions has the negative drawback of affecting social connections, which are the building blocks for adaptive living and timely recovery after stressful experiences (Bonanno et al., 2007). As a result, the levels of loneliness, as well as the levels of depression and anxiety, have increased during the COVID-19 pandemic (Luo et al., 2012; Killgore et al., 2020; Palgi et al., 2020). Young people are particularly vulnerable to depression, anxiety, and loneliness (Groarke et al., 2020), which has been exacerbated by the COVID-19 pandemic (Liu et al., 2020). Thus, the accumulation of negative life events during this period, have deteriorated mental health and could increase the risk of underperformance under the current scenario (Brehl et al., 2021). Surprisingly, however, Gonzalez et al. (2020) found that the confinement has affected academic performance of Spanish students positively. They attributed this effect to changes in study behavior that make students work more continuously and uninterrupted. Also, they might have been relying on an increased feeling of intrinsic responsibilities when facing the new and unknown scenario that the confinement procedures formed. Thus, while for some students the lack of distraction may be helpful in terms of focus and academic performance, it is also conceivable that study performance decreases along with a deterioration of mental health. It could very well be that the coping mechanism employed by students plays a key role in determining the effect on academic performance. A widely used definition of coping is “constantly changing and behavioral efforts to manage specific external and/or internal demands that are appraised as taxing or exceeding the resources of the person” (Lazarus and Folkman, 1985, p. 141). To respond to stressful situations and adapt to changing environmental conditions, individuals can use different coping strategies. Coping strategies have been classified depending on their functionality to solve or evade the challenges: adaptive coping behaviors lean toward acknowledging the stressors, analyzing the situations, seeking support, and making efforts to solve the problems. In contrast, maladaptive coping behaviors lean toward avoiding the problems, withdrawal, and substance use to evade the sources of stress (Folkman and Moskowitz, 2004). However, higher order coping dimensions appear to be unstable and depend on the type of stressors and the sample (Campos et al., 2004).

A widely used scale to measure coping strategies is the Brief COPE (Carver, 1997). The Brief COPE is a shortened version of the COPE inventory (Carver et al., 1989), and it measures 14 different coping responses. However, there is not much consistency in the aggregation of coping strategies to form higher-order factors in the literature, as clusters up to nine factors have been found (Carver, 1997). Originally, coping strategies were categorized into two dimensions, namely problem-focused and emotion-focused coping, clustering into the emotion-focused factor strategies such as venting, behavioral avoidance, and substance abuse. Meanwhile, problem-focused coping strategies have included positive framing, planning, and active coping (Folkman and Lazarus, 1980; Moore et al., 2011). However, categories containing different strategies have been defined in other contexts. Later, a third dimension, maladaptive coping, was added to the previous two (Folkman and Moskowitz, 2004). This third dimension changed the configuration. Problem-focused strategies now included strategies such as taking actions and getting instrumental support, emotion-focused strategies included emotional support, acceptance, positive framing, and the use of religious beliefs, and finally, maladaptive strategies included behaviors such as denial, venting, substance use, and self-blame. However, in previous research it is suggested that the effectiveness of different coping responses is context-dependent (Lazarus and Folkman, 1985; Lazarus, 2000; David et al., 2006). Considering that in previous literature higher-order strategies, such as adaptive and maladaptive coping have found to be composed of different lower-order coping strategies depending on the context (Krägeloh, 2011), it would be advisable to run separate factor analyses and determine the factor structure in the context of the COVID-19 crisis, rather than relying on previously defined factors.

Indeed, prior research showed that coping is multidimensional, and it has been shown that it has both positive and negative dimensions. A negative dimension, categorized as maladaptive coping, which includes avoidant and emotion-focused strategies, and a positive dimension, categorized as adaptive coping, which includes active and problem-focused strategies (Groth et al., 2019). Even though in the past different models high-order factors have not been consistent (Snell et al., 2011), in general they have been consistent in their association to physical and mental health outcomes. Coping strategies have been found to play a role in predicting mental health outcomes over time (Thompson et al., 2018). While adaptive coping strategies such as planning and acceptance aid in solving the problems at hand, maladaptive coping fails to resolve stress sources, causing anxiety, and depression (Lazarus and Folkman, 1985). Another study found that maladaptive coping was a significant predictor of depression, anxiety, and stress, whereas adaptive coping did not significantly predict any of these variables among students (Mahmoud et al., 2012). In particular, maladaptive coping strategies have been linked to higher levels of depression (Heffer et al., 2014), and maladaptive emotion regulation strategies to anxiety during the COVID-19 crisis (Brehl et al., 2021). On the other hand, adaptive and instrumental or social coping strategies have been associated with better stress management and reduced negative mental health outcomes (Farley et al., 2005). In general, maladaptive coping strategies, such as withdrawal and substance abuse, seem to be adopted and increase in early adolescence, stabilizing at the end of the school years (Skinner and Saxton, 2019). The mechanism by which maladaptive coping strategies are likely to impact mental and academic outcomes is by preventing individuals from facing and solving the problems. As Beck and Clark (1997) proposed, negative mental outcomes are not directly caused by the stressors themselves, but by the individuals’ perceptions of and reactions to those stressors. Thus, the outcomes are more a consequence of the individuals’ skills to interpret and cope with the stressors. In this context, coping strategies could serve as tools to understand differing responses to challenges to manage stressful situations (Folkman and Moskowitz, 2004). Although the relationship between emotion-focused coping strategies and academic performance has been stablished (Thomas et al., 2017), we predict that this relationship is mediated by procrastination, due to its consequences on delaying taking actions upon challenges. This may be related to a negative downward spiral, in which people also develop a tendency to procrastinate.

Procrastination is defined as “the voluntary delay of an intended and necessary and/or (personally) important activity, despite expecting potential negative consequences that outweigh the positive consequences of the delay” (Klingsieck, 2013, p. 26). It has been found to be more prevalent in people with poor mental health (Stead et al., 2010), and has been associated with decreased academic performance (Kim and Seo, 2015; Tice and Baumeister, 2018). This impact on performance could be caused by assigning less time for working on the tasks (Buehler et al., 1994), not assigning time to act upon unforeseen obstacles, or an impaired performance caused by working under stressful situations (Baumeister, 1984). Students are particularly prone to procrastination, as prior research showed up to 50% of students perceived it as a cause of distress and difficulties (Day et al., 2000). Prior research has found a relation between procrastination and depression, and it has been theorized that it might be an outcome rather than a predictor of depressive mental states (Martin et al., 1996; van Eerde, 2003; van Eerde and Klingsieck, 2018). Procrastination is also related to higher levels of anxiety in students (Haycock et al., 1998), and it has been proposed that both anxiety and depression would make students more vulnerable to repetitive negative thoughts about past events, which in turn increases levels of procrastination (Constantin et al., 2018).

Whereas some studies have examined the relation between loneliness, anxiety, and depression, other mechanisms that facilitate the negative trend and their impacts on academic performance have often been overlooked. The current research aims to integrate these factors into a more comprehensive model in the context of the COVID-19 crisis while also incorporating the overlooked outcomes on academic performance by searching for a mechanistic explanation of the role of coping strategies and procrastination on these outcomes (see Figure 1). A better understanding of the role of individuals’ strategies to respond to stressful situations and to perform academically would help improve students’ tools to adapt to these situations and improve mental health and academic performance.

In the present study, two steps will be taken. First, we analyze the factor structure of the coping strategies during the COVID-19 pandemic. Based on previous literature findings, we expect to find at least two higher order coping factors, one positive and one negative. Second, we use longitudinal data to test a sequential mediation model to examining how the COVID-19 crisis affects psychological well-being and academic performance of first-year university students, as well as the mediating role of coping strategies and loneliness in this process. Furthermore, we examine the mediating role of procrastination between psychological well-being and academic performance.

Figure 1 gives an overview of the model and hypotheses tested in the current study. Based on previous findings we hypothesized a sequential model in which psychological well-being before the COVID-19 pandemic (T1) is related to perceived loneliness and coping strategies that students used at the beginning of the COVID-19 crisis (T2). These coping strategies and perceived loneliness should subsequently predict psychological well-being later in the academic year (T3), which might affect academic performance at the end of the academic year (T4), possibly mediated by procrastination (T3).

**Hypothesis 1:** Individuals with higher levels of anxiety and depression at T1 will have of higher levels of loneliness at T2.

**Hypothesis 2:** Individuals with higher levels of anxiety and depression at T1 will rely on more negative coping strategies at T2.

**Hypothesis 3:** Loneliness at T2 will be positively associated with anxiety and depression at T3.

**Hypothesis 4:** Maladaptive coping strategies at T2 will be positively associated with depression and anxiety at T3.

Anxiety and depression have been associated with higher levels of procrastination (Steel, 2007). Hence the following hypothesis:

**Hypothesis 5:** Higher levels of anxiety and depression will be associated with higher levels of procrastination at T3.

Considering the consequences of procrastinating behaviors and the irrational delay in initiating important activities, we also test the following hypothesis:

**Hypothesis 6:** Procrastination at T3 will be negatively associated with academic performance at T4.

Furthermore, we examine the mediating role of loneliness and maladaptive coping between psychological well-being at T1 and T3 with the following hypothesis:

**Hypothesis 7:** Loneliness and maladaptive coping at T2 partially mediate the relationships between anxiety and depression at T1 and anxiety and depression at T3.

Based on earlier found relationships between psychological well-being, procrastination, and academic performance, we also test the following hypothesis:

**Hypothesis 8:** Procrastination at T3 mediates between anxiety and depression at T3, and academic performance at T4.

Finally, if Hypotheses 1–8 are supported in the degree that it is possible to find sequential significant relations from the initial assessments to the outcome, it would suggest the following hypothesis:

**Hypothesis 9:** Loneliness and the negative coping strategies at T2, as well as anxiety, depression, and procrastination at T3 sequentially mediate between anxiety and depression at T1 in the initial stages of the COVID-19 pandemic, and academic performance at T4, at the end of the semester.

## Materials and Methods

### Participants

Participants were 881 first year bachelor students of a Business Administration (BA) bachelor’s degree program at a Dutch university. A total of 502 students followed the Dutch BA track, and 379 students were in the International Business Administration (IBA) track. The average age of the students was 18.58 years (SD = 1.28), and 59.7% was male. The curriculum was the same for both groups, except the language (Dutch or English). In consideration that the differences between groups were small in comparison with the scales’ range (see Supplementary Table A), and that the results did not change significantly when analyzed in separate, the two groups were combined into one sample. When we repeated our main analyzes for the two groups separately, the results were also highly similar. The original number of students who participated in the program was 1533. We gave all students an informed consent at the start of the study. Of 395 students, consent data was incomplete, or they did not give consent, and 257 students did not complete any of the surveys that were needed for the present study. Therefore, our final sample for research purposes consisted of 881 students. Out of the 881 students in the total sample, 824 students responded to the questionnaires at T1 (468 BA and 356 IBA), 630 at T2 (326 BA and 304 IBA), and 460 at T3 (247 BA and 213 IBA).

### Ethics Statement

The study was approved by the Internal Research Board of the university. Longitudinal data collection took place during a professional development course, which was a mandatory part of the curriculum. However, students were given the option to opt-out from having their data used for research purposes by either indicating this on their informed consent form, or by sending an email to the research team at any time during the study.

### Procedure

**FIGURE 2:**
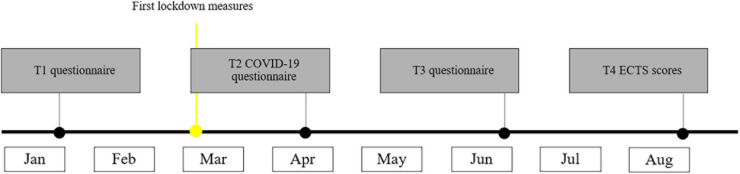
A timeline of the measurements.

The current study was part of a larger longitudinal project. Data collection took place in the context of a course focused on professional development that all first-year students followed throughout the academic year 2019–2020. The study originally comprised online surveys of several questionnaires across the academic year, that assessed psychological constructs, including depression, anxiety, and procrastination. In the second half of the academic year, the COVID-19 pandemic reached the Netherlands, and from March 2020 onward, restrictive measures were being taken to contain the virus. These included working from home and school and university closures. Students were given lectures online. For international students, this often meant they could not return to their home country during this time. In April, an extra questionnaire was sent out electronically to the students to assess how they were handling the situation. In this questionnaire, coping strategies and loneliness were assessed. Thus, data collection for the analyses of the current paper occurred over a 7-month period during the second half of the academic year (January to August). Figure 2 provides a timeline of the different measurement occasions that were included in the present study.

### Measures

#### Depression

Depression (T1 and T3) was measured using the Patient Health Questionnaire (PHQ-9), a short questionnaire aimed at assessing the severity of depression (Kroenke et al., 2001). Participants were asked to indicate how often over the last 2 weeks they had been bothered by nine different symptoms of depression such as “feeling down, depressed, or hopeless,” or “feeling tired or having little energy.” A 4-point scale was used, ranging from not at all to nearly every day. Cronbach’s alpha was 0.87 at T1, and 0.9 at T3.

#### Anxiety

To measure anxiety (T1 and T3), we used the GAD-7, a brief seven-item questionnaire to assess generalized anxiety disorder (Spitzer et al., 2006). Like for depression, participants were asked to indicate how often over the last 2 weeks they had been bothered by seven different symptoms of anxiety, such as “Feeling nervous, anxious, or on edge,” or “worrying too much about different things.” The questions were answered on a 4-point scale, ranging from not at all to nearly every day. In the current sample, Cronbach’s alpha was 0.9 at T1, and 0.94 at T3.

#### Loneliness

Students’ experienced level of loneliness since the start of the COVID-19 crisis was assessed at T2 (about a month after the start of the lockdown measures) using the six-item De Jong Gierveld Loneliness Scale (Gierveld and van Tilburg, 2006). This is a short version of the earlier 11-item De Jong Gierveld Loneliness Scale (de Jong-Gierveld and Kamphuls, 1985), which includes statements such as “I miss having people around,” and “I miss having a really close friend.” The six-item version has been shown to have good reliability and validity in previous research (Gierveld and van Tilburg, 2006). In the present study, the Cronbach’s alpha was 0.68 (T2).

#### Coping With the COVID-19 Crisis

To measure coping, we used the Brief COPE (Carver, 1997) also at T2. The Brief COPE is a shortened version of the COPE inventory (Carver et al., 1989). It assesses 14 different coping responses (self-distraction, active coping, denial, substance use, use of emotional support, use of instrumental support, behavioral disengagement, venting, positive reframing, planning, humor, acceptance, religion, and self-blame), using two items per scale.

Literature suggests that the Brief COPE can be used to measure situational or dispositional coping (Carver and Scheier, 1994). Because of the specific and unusual circumstances, and because we were interested in how student cope with the current situation, we measured situational coping, targeted at the COVID-19 situation. The questionnaire was introduced with the instructional sentence “Since the start of the corona crisis, ….” The subscale self-blame was omitted, as the items did not seem suitable within the COVID-19 situation, as it seemed highly unlikely that students would blame themselves for the developed situation. Items were answered on a 5pt scale (not at all – a lot). In line with the recommendation by Eisinga et al. (2013) we assessed the reliability of the two-item (sub)scales using the Spearman–Brown coefficient rather than Cronbach’s alpha as the later provides a less accurate estimate of reliability for two-item scales. Reliabilities of all subscales are shown in Table 1.

**TABLE 1:** Reliability of the scale scores for the Brief COPE.

**Brief COPE subscale**	**Spearman–Brown coefficient**
Self-distraction	0.20
Active coping	0.47
Denial	0.79
Substance use	0.85
Emotional support	0.80
Behavioral disengagement	0.43
Venting	0.50
Instrumental support	0.74
Positive framing	0.71
Planning	0.62
Humor	0.70
Acceptance	0.65
Religion	0.81

#### Procrastination

Procrastination (at T3, 3 months after the start of the lockdown and 2 months after the T2 questionnaire) was assessed with the Avoidance Reactions to a Deadline Scale (van Eerde, 1998), which includes items such as “I begin later than I had planned,” and “I say to myself start now. And I still don’t start.” This scale consists of eight items that measure cognitive and behavioral acts when students are confronted with deadlines. Items were answered on a 5-point scale (Never – Always). The Cronbach’s alpha in the current study was 0.88 at T3.

#### Academic Performance

Official university transcripts were collected for all participants after the first year (T4) in order to derive the number of obtained European Credit Transfer and Accumulation System or ECTS credits (0–60). One successful academic year corresponds to 60 ECTS credits, which translates to 1500–1800 study hours. In order to earn ECTS credits for a course, a student needs to achieve a passing mark for each course. The scale for the grades for each course varied from 1 to 10, with 1 meaning bad and a 10 being excellent. Students with a grade lower than 5.5 failed the course. This measure of academic performance is most predictive of later academic performance and proved to be a reliable predictor of incremental study progress in the European context (e.g., Schippers et al., 2013, 2015, 2020; Triventi, 2014).

### Analytical Procedure

Prior to our main analyses, we conducted a second-order exploratory factor analysis (EFA) on the subscales of the Brief COPE in order to explore the different coping strategies in the context of the COVID-19 crisis. Using confirmatory factor analysis (CFA) we validated the resulting higher-order coping strategies. Once the best fitting factor solutions had been identified, the resulting higher-order factors were incorporated in our theoretical model (Figure 1). In order to test our main hypotheses, structural equation modeling (SEM) was applied to test the partially mediating role of the different higher-order coping strategies and loneliness on the relationship between depression and anxiety at T1 and T3. In addition, the model included the fully mediating role of procrastination on the relationship between depression and anxiety at T3 and academic performance at the end of the academic year (T4). The Lavaan package (0.6–7) in R was used (Rosseel, 2012) for the modeling procedure. To judge the model fit, different fit indices are reported (χ^2^-value, χ^2^/df ratio, CFI, RSMEA, and SRMR) following the recommendations of Schreiber et al. (2006) regarding the cut-off criteria. To avoid biased estimates as a result of non-response and in order to improve the validity of the statistical results, the modeling procedure made use of a “Full information maximum likelihood estimation” (FIML) for handling missing values (Enders and Bandalos, 2001). Note that missing data due to non-response on the different questionnaires was found to be either missing at random (MAR) or completely missing at random (MCAR) after eliminating outliers. To confirm the if the database met the MAR criteria, a regression-based approach using the RBtest library from R was run (Rouzinov and Berchtold, 2020). To test the statistical significance of the mediation effects (see section “Results”) we additionally calculated the 95%-confidence interval (CI) with bias corrected standard errors using a bootstrapping approach with 5000 repetitions (Shrout and Bolger, 2002; Kline, 2015).

## Results

### Descriptives

The means, standard deviations, and correlations for all variables and measurement occasions are displayed in Supplementary Material. Examination of the residual plots revealed that the assumptions of linearity and homoscedasticity were met for the observed variables. The standard deviation, mean scores, and bivariate correlations between all variables at each measurement occasion are presented in Supplementary Table B.

### Outliers

Prior to the modeling procedure the data were scanned for possible multivariate outliers. To identify multivariate outliers the Mahalanobis distance was calculated, with a criterion of *p* < 0.001, which led to the detection of 98 multivariate outliers which were excluded from the analysis, leaving the total sample in 783 observations. However, the vast majority of those cases was detected due to missing data on multiple constructs. When calculating the Mahalanobis distance for only those cases with complete data on all variables and measurement occasions only 13 cases were found to be outliers.

#### Factor Structure of Brief COPE

In order to reduce the number of factors and create a more parsimonious model, we performed a second-order EFA. The results of this EFA are presented in Table 2. As expected, and in line with the literature, three distinct second-order factors with an eigenvalue over 1 were identified.

**TABLE 2:** Summary of the second-order exploratory factor analysis of the coping strategies.

**COPE subscales**	**Factor 1**	**Factor 2**	**Factor 3**
Planning	**0.682**	0.321	0.05
Positive framing	**0.63**	0.165	−0.043
Active coping	**0.56**	0.143	−0.077
Self-distraction	0.361	0.170	0.124
Instrumental support	0.222	**0.951**	0.204
Emotional support	0.365	**0.653**	0.115
Denial	0.123	0.071	**0.67**
Behavioral disengagement	0.015	0.092	**0.601**
Venting	0.249	0.321	**0.52**
Substance use	−0.014	0.052	**0.452**
Non-acceptance	0.344	0.039	**0.475**
Humor	0.281	0.012	0.102
Religion	0.108	0.131	0.162

**n* = 623. *Rotated f*actor loadings over 0.40 appear in bold. Eigenvalues after the extraction = 1.063. Percentage of variance 38.8%.*

Based on the criteria of factor loadings above 0.4 (Guadagnoli and Velicer, 1988), the resulting factors consisted of the following sub-scales. Factor 1, adaptive coping, includes the scales of positive framing, planning, and active coping. Factor 2, supportive coping, includes the scales of instrumental support and emotional support. Factor 3, maladaptive coping, includes the scales of denial, behavioral disengagement, venting, substance use, and acceptance (reverse coded). Humor and religion did not load above 0.3 in any of the three predicted factors. Therefore, these scales were left out of the analyses. The acceptance sub-scale was reverse coded because its highest load was −0.475 in Factor 3. By doing this, we made sure that all the factor loadings for each sub-scale were in the same direction, and hence this scale represents “non-acceptance.”

Although some of the first-order scales showed reliabilities below the recommended value of 0.7 (Nunnally, 1994), we considered the issues that have been found with two item scales (Eisinga et al., 2013) and assessed the composite reliability of the second-order factors, including all the scales with a factor loading above 0.4. We found that each of the three factors reached an acceptable-to-good Cronbach’s alpha. For Factor 1 Cronbach’s alpha was 0.76, for Factor 2, 0.86, and for Factor 3, 0.75. Note that none of the composite reliabilities of the three factors would have improved if any of the included subscales were dropped from their respective second-order factors.

Next, a CFA was applied to validate the results of the EFA. For this analysis, we included only those subscales that loaded high on any of the three distinct factors that were found in the EFA.

Using the maximum likelihood method, we estimated the model fit using the χ^2^/df ratio, CFI, RMSEA, and SRMS. The models’ *p*-value was not considered, as the typical Chi-square for large samples is not significant. To analyze the fit of the models, we used the criteria of CFI > 0.90, RMSEA < 0.08, SRMR < 0.08, and χ^2^/df < 3 (Hooper et al., 2008; Iacobucci, 2010).

**FIGURE 3:**
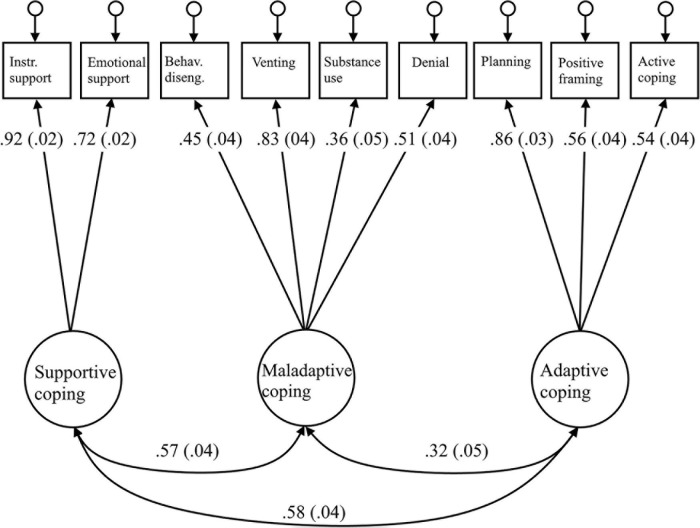
Three-factor solution for coping. Behav. diseng, behavioral disengagement; Instr. support, instrumental support. All loadings are significant at *p* < 0.05. Standard errors are reported in parentheses. Model fit: χ^2^ = 70.66, χ^2^/df = 3.21, CFI = 0.967, RMSEA = 0.06, and SRMR = 0.047.

An initial test of the three-factor model indicated a less than acceptable fit with the following parameters: χ^2^ = 238.89, χ^2^/df = 7.46, CFI = 0.875, RMSEA = 0.102, and SRMR = 0.087. The examination of the standardized residuals and modification indices suggested adjusting the model by incorporating the non-acceptance factor as a latent variable to support coping and adaptive coping. As the loading factor of non-acceptance in the CFA was low (0.34), and the loading of a given variable in more than one factor is not advisable for structural models, we eliminated the non-acceptance variable from the model. The new model resulted in: χ^2^ = 109.11, χ^2^/df = 4.54, CFI = 0.943, RMSEA = 0.075, and SRMR = 0.053, showing a significant improvement in all the criteria. Finally, a new examination of standardized residuals and modification indices suggested to permit the correlation of errors between emotional support – positive framing and denial – behavioral disengagement. This definitive model resulted in: χ^2^ = 70.66, χ^2^/df = 3.21, CFI = 0.967, RMSEA = 0.06, and SRMR = 0.047, meeting all the fitting criteria. The results of the three-factor solution are presented in Figure 3.

The definitive three-factor model reached a much better fit than the two-factor model considering problem-focused and emotion focused coping: χ^2^ = 810.307, χ^2^/df = 15.28, CFI = 0.573, RMSEA = 0.151, and SRMR = 0.120, or the three-factor model that also distinguishes dysfunctional coping: χ^2^ = 541.06, χ^2^/df = 10.6, CFI = 0.724, RMSEA = 0.124, and SRMR = 0.097.

To conclude, the three second-order factors, denominated adaptive, supportive, and maladaptive coping, were used in the next steps of our analyses, confirming our expectation of finding at least one negative and one positive higher order factor.

To conclude, the three second-order factors, denominated adaptive, supportive, and maladaptive coping, were used in the next steps of our analyses, confirming our expectation of finding at least one negative and one positive higher order factor.

#### Sequential Model

For the modeling procedure we employed a model generation approach as outlined by Kline (2015). In that fashion we tested out initial theoretical model but allowed for several iterations of further model specification in the case of unsatisfactory model fit. The respecified model was then tested again with the same data. As described by Kline (2015), while not strictly confirmatory in the narrowest sense, the model generation approach is the most common approach and follows the goal of finding a model that makes theoretical sense, is reasonably parsimonious and in close correspondence to the data.

Following this approach led to four iterations of model re-specification. The resulting five models are described subsequentially in the following section. The fit coefficients for all five models are presented in Table 3.

**TABLE 3:** Model fit coefficients for the structural equation models.

	**χ^2^**	**χ^2^/df**	**CFI**	**RMSEA**	**SRMR**
Model 1	664.98	7.47	0.755	0.095	0.120
Model 2	421.90	7.81	0.800	0.097	0.0103
Model 3	404.59	7.35	0.810	0.094	0.094
Model 4	181.55	5.18	0.887	0.076	0.062
Model 5	90.81	2.67	0.971	0.046	0.040

##### Model 1 – the initial model

Our initial model (Figure 1) consisted of two exogenous variables at T1, anxiety and depression, modeling the partial mediation of loneliness and the three coping factors identified during the CFA, and the resulting anxiety and depression variables measured at T3. This original model also included the mediating role of procrastination between depression and anxiety at T3, and the academic outcomes as number of credits approved at the end of the semester. Due to the complexity of the model only the higher-order coping strategies were estimated as latent factors. For all other constructs in the model the mean scores were used as manifest variables. This considerably reduced the number of parameters that had to be estimated and allowed for model convergence. The initial model tested the mediating role of loneliness and the three coping strategies on the levels of anxiety and depression from T1 to T3 as well as the mediating role of procrastination in the relationship between depression and anxiety at T3, and study performance at T4. Model 1 resulted in non-significant paths for adaptive coping, and for the direct relation between anxiety at T3 and procrastination at T3. The model did not reach any acceptable fit indices at this point (χ^2^/df = 7.47, CFI = 0.755, RMSEA = 0.095, SRMR = 0.120).

##### Model 2

The second model eliminated all the non-significant adaptive coping paths from the analysis, resulting in a model where the supportive coping factor lost significance in the depression path. The model did not reach any acceptable fit indices at this point (χ^2^/df = 7.81, CFI = 0.800, RMSEA = 0.097, SRMR = 0.103). Modification indices suggested to allow the error terms of denial and behavioral disengagement to covariate. As they were measured at the same time, and are part of the same latent variable, it was added to model 3.

##### Model 3

The third model eliminated the non-significant path of supportive coping in the depression path, resulting in a model where the supportive coping lost significance in the anxiety path. The model did not reach any acceptable fit indices at this point (χ^2^/df = 7.35, CFI = 0.810, RMSEA = 0.094, SRMR = 0.094). Modification indices suggested to allow the error terms of maladaptive coping and loneliness to covariate. As they were measured at the same time, it was added to model 4.

##### Model 4

The fourth model eliminated the non-significant path between anxiety and supportive coping, eliminating all the contributions of supportive coping and consequently removed the factor from the model. The model did not reach any acceptable fit indices at this point (χ^2^/df = 5.18, CFI = 0.887, RMSEA = 0.076, SRMR = 0.062). Modification indices suggested to allow the error terms of depression and anxiety to covariate. Thus, as they were measured at the same time, depression and anxiety at T1, and at T3 were allowed to covariate and added to model 5.

##### Model 5

**FIGURE 4:**
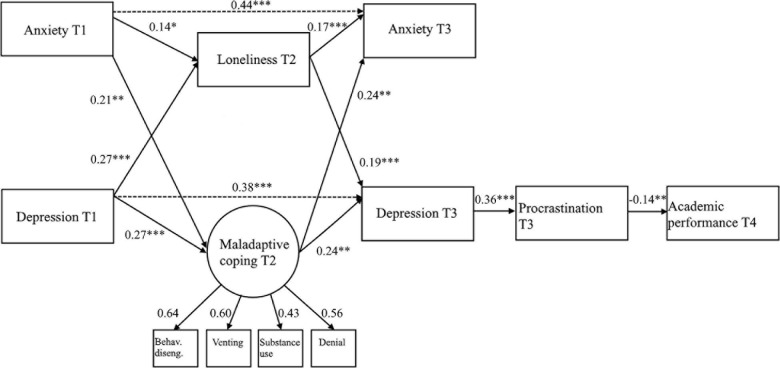
Final model (model 5) with path coefficients and factor loadings. Behav. diseng, behavioral disengagement. Values represent standardized path coefficients. Covariances and error terms not included for clarity ^∗^*p* < 0.05; ^∗∗^*p* < 0.01; ^∗∗∗^*p* < 0.001.

In order to create a more parsimonious model, the fifth model kept all the significant paths between depression, anxiety, loneliness, maladaptive coping, procrastination, and academic performance, eliminating all non-significant paths among them. This model reached excellent fit indices (χ^2^/df = 2.67, CFI = 0.971, RMSEA = 0.046, SRMR = 0.040), and its path coefficients are shown in Figure 4.

The results obtained in model 5 confirm our expectation for Hypotheses 1 and 2, as higher levels of anxiety and depression at T1 were found to be related with higher levels of loneliness (H1) and maladaptive coping (H2) at T2. Hypotheses 3 and 4 were also supported, as both loneliness (H3) and maladaptive coping (H4) at T2 were significantly associated with higher levels of anxiety and depression at T3. For Hypothesis 5, the expectation was only partially supported, as only depression, but not anxiety at T3 was significantly correlated with procrastination at T3. As the final step in checking the individual relations, Hypothesis 6 was also supported by the negative but significant relation between procrastination at T3 and academic performance at T4.

When looking at the mediation hypotheses, results generated by 5000 iterations of bootstrapping (Shrout and Bolger, 2002) indicate that loneliness at T2 partially mediated from depression at T1 to depression at T3 (indirect effect = 0.054, bias-corrected bootstrap 95%-CI 0.022–0.101), and from anxiety at T1 to anxiety at T3 (indirect effect = 0.025, bias-corrected bootstrap 95%-CI 0.005–0.061). Maladaptive coping at T2 partially mediated from depression at T1 to depression at T3 (indirect effect = 0.069, bias-corrected bootstrap 95%-CI 0.018–0.161), and from anxiety at T1 to anxiety at T3 (indirect effect = 0.053, bias-corrected bootstrap 95%-CI 0.011–0.135). Thus, it was supported that loneliness and maladaptive coping at T2 partially mediate between anxiety and depression at T1 and T3 (H7), even in the presence of a direct path between anxiety and depression at T1, and anxiety and depression at T3. The second mediation hypothesis test also resulted in a significant mediation of procrastination at T3, between depression at T3 and Academic performance at T4 (H8). Although unexpected, the lack of significance in the relation between anxiety and procrastination could be explained by the high correlation between anxiety and depression. This assumption was confirmed by assessing an alternative model that tested the significance of the relation between anxiety and procrastination at T3 in the absence of depression at T3, which resulted in a significant positive correlation. However, this model had worse fit that the definite model 5 and for this reason we decided to keep the model that fully mediates academic performance at T4 and depression at T3 *via* procrastination at T3.

Finally, as Hypotheses 1–8 were supported, allowing to find significant sequential relationships from the initial assessments at T1 to the outcome of academic performance at T4, we found significant support for the hypothesis that loneliness and negative coping strategies at T2, as well as depression and procrastination, but no anxiety at T3 sequentially mediate between anxiety and depression at T1 and academic performance at T4 during the COVID-19 pandemic (H9).

## Discussion

Coping with difficult situations is highly important in all situations and become even more important during the COVID-19 crisis. In the current study we showed that maladaptive coping strategies and loneliness play a significant role in the trajectory of mental health outcomes, and how these outcomes impact on academic outcomes through the mediation of procrastination in undergraduate management students during the COVID-19 pandemic. Maladaptive coping strategies in the form of denial, substance use, behavioral disengagement, and venting increased the levels of anxiety and depression after the lockdown restrictions started. Higher levels of depression, in turn, increased the levels of procrastination, impacting academic performance negatively.

The results of this study extend the findings in the literature, offering a theoretical explanation for the role of coping strategies, loneliness, and procrastination in the trajectory of anxiety and depression over time during the COVID-19 pandemic. First, we found supporting evidence of a high order three-factor configuration for coping styles using the Brief COPE questionnaire, identifying three groups of coping strategies that we denominated adaptive coping (planning, active coping, and positive framing), supportive coping (emotional and instrumental support), and maladaptive coping (denial, substance use, behavioral disengagement, and venting). In addition, the fact that the higher-order factor (supportive coping) combined emotional and instrumental support was in line with the studies by Carver (1997) and Farley et al. (2005), concerning the Brief COPE, and Carver et al. (1989), concerning the original COPE scale. Second, we tested the role the three high-order factors of coping in the trajectories of mental health outcomes, finding that only maladaptive coping partially mediates the subsequent changes in the levels of depression and anxiety. Third, the results reveal that loneliness during the pandemic is a significant partial mediator of the subsequent changes in the levels of depression and anxiety. Fourth, the study shows that procrastination is a significant mediator between the later levels of depression and the academic performance of students.

The lack of significance of adaptive and supportive coping with depression and anxiety during the COVID-19 pandemic suggests that the most important drivers for negative mental health outcomes are the maladaptive coping behaviors, which is in line with the findings by Groth et al. (2019), who found that maladaptive, and not adaptive coping was significantly associated with anxiety and depression during “normal times.” The significant relationship from maladaptive coping to depression shows that people that engage in challenge-evading behaviors end up with higher levels of depression. Indeed, the dimensions that form maladaptive coping have broad implications in acting on non-contributing manners to adjust and respond to emotional stressors, offsetting the adaptive and supportive coping strategies’ potential positive contributions. Thus, interventions aimed at reducing depression and anxiety among students should emphasize reducing maladaptive coping strategies to modify the negative consequences of maladaptive behaviors.

The role of loneliness as a mediator for anxiety and depression confirms that the mismatch between expected and real social connection can cause higher levels of negative mental outcomes (Palgi et al., 2020). This outcome enhances the necessity to focus on different options to prevent social isolation and increase the awareness of the benefits of social connections. In the context of COVID-19 some of the options could be to make restrictions of social gatherings more flexible if the health prevention measures can be implemented, or to promote the creation of “safe social bubbles” where a group of people with more controlled external interactions can gather with a limited number of people as long as everybody respects the health-preventive recommendations (Leng et al., 2020).

In line with previous findings from DeRoma et al. (2009) and Owens et al. (2012), we found that higher levels of depression are significantly associated with lower academic performance, and that procrastination is a significant mediator of this relationship. Thus, it could very well be that higher levels of depression would trigger more procrastination, postponing the initiation of duties and impacting the final quality of the academic performance. This is particularly important in the context of the COVID-19 crisis, as the levels of depression have increased up to sevenfold (Bueno-Notivol et al., 2021). Among the two mental health outcomes, only depression was found to be significantly correlated with academic outcomes, and this association is significantly mediated by procrastination. At first glance, this suggests that depression and not anxiety prevents people from taking effective actions toward a better performance. As discussed by Eysenck et al. (2007), anxiety may not always result in impaired performance. Besides reducing cognitive control, anxiety also heightens attention to threat stimuli, which in the presence of compensatory strategies like increase in effort and process resource could prevent negative outcomes. However, the analysis of an alternative model in our study shows that in the absence of depression, anxiety can also significantly predict procrastination and lower academic performance, which is consistent with the literature that found that both anxiety and depression can contribute to procrastination (Haycock et al., 1998; Constantin et al., 2018).

Our study and research model integrate the knowledge of the inter-relation between loneliness, coping strategies, procrastination, mental health outcomes and academic performance during the COVID-19 crisis. The advantage of creating a model using SEM is that it allows to perform direct tests of the theory while giving some control of the measurement errors. The resulting structural model offers possible intervention targets at different stages. Based on the longitudinal nature of the observations, it would be theoretically possible to improve the mental health outcomes of depression and anxiety by intervening in the maladaptive coping strategies and the level of loneliness before the appearance of negative mental health outcomes. On the other hand, to improve the academic outcomes, it would be possible to intervene in an early stage to reduce the perceived levels of loneliness and the maladaptive cooping behaviors, or in a later stage, by targeting students’ procrastination habits.

## Strength, Limitations, and Future Directions

An important strength of this study is that we used a large sample and that we started our surveys before the first lockdown occurred. Moreover, we measured our relationships using a longitudinal design, including measuring anxiety and depression at more than 1 point in time while measuring academic performance at the end of the academic year. The combination of a repeated measurements design, and the events in response to the COVID-19 pandemic have allowed us to work with a robust model to better understand the relationships between negative mental health outcomes, loneliness, coping strategies, and academic performance in students.

Despite the advantages of using large and longitudinal samples and a method that allows to test complex theories it should be mentioned that the causal inferences derived from the structural equation models do not include a control group. For obvious reasons, we were not able to test how a group of students would fare without a lockdown. Another limitation is that except for academic performance, all our data relies on self-reports. Even though our sample included a wide representation of international and Dutch students, it was limited to students in the university population. Future research could examine the relative importance of these variables analyzed in a context other than higher education, other crises, and possibly also in relatively “normal” times, and not in a crisis context. Although a longitudinal design has many advantages, a notable disadvantage is the risk of non-response of the participants at any point of the study, which could threaten the validity of the results. In this case, although there was a reduction in the number of participants from T1 to T3, the sample met the “Missing At Random” criteria (Seaman et al., 2013), which allows handling missing data in a statistically valid manner and allows using the FIML to produce unbiased estimates using the data of participants that did not complete all three surveys (Enders, 2013).

The overall result of a non-significant role of adaptive and supportive coping strategies is not surprising, as other studies have found that maladaptive coping and not adaptive or supportive coping are mediators of negative mental health outcomes (Groth et al., 2019). Still, it does not mean that they don’t have a role in academic outcomes, but the effect is not *via* a path of depression and anxiety. Based on prior literature, we would expect that adaptive and supportive coping play a more significant role in the context of happiness and/or more positive mental health outcomes (Meyer, 2001). Future studies could explore the mediating role of adaptive and supportive coping, both, in the COVID-19 and other contexts. Future research could also focus on ways to enhance more adaptive and supportive coping. For instance, interventions that trigger more positive forms of coping, based on the broaden and build theory (Fredrickson, 2001, 2004), might help. The broaden and build theory posits that experiences of positive emotions can increase the repertoire of thought-actions in individuals, also building their personal resources skillsets, including physical, intellectual, social, and psychological skills. In practice, it may help people come out of a downward spiral of negative emotions, and more into an upward spiral of positive emotions (Schippers and Ziegler, 2019). A promising intervention in this respect is life crafting, a process in which people actively reflect on different aspects of their current and future life and undertake actions to change the areas in a way that aligns with their passions, values, and wishes (Schippers and Ziegler, 2019; de Jong et al., 2020). In this online and scalable intervention, students write about all aspects of their life, and they are asked to write about the ideal life if there were no constraints and to contrast this with their expected life if nothing changes. In a second part of the life crafting intervention, they are asked to make concrete plans, order these plans from most important to least important and to make back-up plans. The advantage of this intervention is that it is inexpensive and scalable to a large number of students. Additionally, an app or chatbot with such an intervention could even add more value in terms of student well-being (Dekker et al., 2020). Other interventions, that could be used in combination with life crafting, are counseling and exercises as means to alleviate symptoms of stress, anxiety, and depression in university students (Byars, 2005).

Having scalable interventions to improve mental health and academic performance is important to reach a large number of individuals. Schippers and Ziegler (2019, p. 12) state that:

“Given the relatively low amount of costs and administrative work that the implementation of the outlined life crafting intervention entails, especially when compared to the potential benefits, we recommend its inclusion in student’s curriculums. Getting many (young) people to take part in an online life crafting intervention may be an important step in achieving not only higher academic performance, but also better well-being, happiness, health, and greater longevity (see Schippers et al., 2015). Using technology to assist with life crafting *via* a goal-setting intervention seems to be a particularly promising avenue as this is an approach that can be easily scaled up.”

These results are important not only for the academic context, as the integration of the role of maladaptive coping strategies in the relationship between loneliness, depression, and anxiety can be beneficial for the research of mental health in any context. The process of engaging in maladaptive coping behaviors appears to have a significant effect in increasing the levels of anxiety and depression.

## Conclusion

In our study we showed how coping strategies and loneliness mediate the changes in depression and anxiety in first year bachelor students, and how in turn, procrastination bridges from these negative mental health outcomes to worse academic performance. We hope that this comprehensive model will help targeting interventions to improve mental health in students, as well as rising awareness about the behavioral mechanisms that impact academic outcomes. We anticipate that focusing on loneliness, maladaptive coping strategies, and procrastination will improve mental health in students while improving academic performance during the COVID-19 crisis. The advantage of an integrative model is that it allows to understand the potential connection between behaviors such as procrastination, coping skills, and academic and mental health outcomes. Understanding how these factors affect each other offers the opportunity to identify individuals that might suffer from depression or anxiety and implement interventions to reduce their perceived levels of loneliness, expecting to reduce their levels of depression and anxiety while increasing their academic performance.

Universities have had to make complicated decisions to transition toward virtual education without having a clear idea of the impacts of these decisions on students’ mental health. This study revealed some of these unintended consequences on their students and provides an opportunity to counteract the negative outcomes, e.g., by providing virtual counseling support (Lee et al., 2021), or explore new ways to promote connection among students (Stuart et al., 2021). Thus, reducing the feelings of loneliness and creating awareness of the importance of effective coping skills. Promising interventions such as life crafting and well-being apps could potentially become part of the student’s curriculum, as the mental health and academic benefits far outweigh the costs (Schippers et al., 2015, 2020; Schippers and Ziegler, 2019; Dekker et al., 2020).

We hope that our findings inspire subsequent studies that open other potential avenues to understand the inter-relation of other positive and negative mental health paths to improved performance in academic and more general settings. The availability of this type of models can serve not only universities, but also governments and policymakers to identify more effective targeted interventions in order to buffer the downstream unintended consequences of health and social policies.

## Data Availability Statement

The raw data supporting the conclusions of this article will be made available by the authors, without undue reservation.

## Ethics Statement

The studies involving human participants were reviewed and approved by the Rotterdam School of Management internal research board. The patients/participants provided their written informed consent to participate in this study.

## Author Contributions

SF wrote the initial draft of the manuscript and ran the majority of the statistical analyses. SF and NZ were involved in the analytical process and, revision and interpretation of the results. SF, NZ, and EJ contributed to the preparation of the raw data and the restructuring and revision the manuscript. MS provided the intellectual feedback and contributed to different versions of the manuscript. All authors were involved in the conception and design of the study, agreed to all aspects of the manuscript, and approved the final version.

## Conflict of Interest

The authors declare that the research was conducted in the absence of any commercial or financial relationships that could be construed as a potential conflict of interest.

## Publisher’s Note

All claims expressed in this article are solely those of the authors and do not necessarily represent those of their affiliated organizations, or those of the publisher, the editors and the reviewers. Any product that may be evaluated in this article, or claim that may be made by its manufacturer, is not guaranteed or endorsed by the publisher.
