# Supplementary material for: Depression and Anxiety in Times of COVID-19: How Coping Strategies and Loneliness Relate to Mental Health Outcomes and Academic Performance
Source: Front Psychol. 2021 Oct 25;12:682684. doi: 10.3389/fpsyg.2021.682684 (PMC8572913; doi:10.3389/fpsyg.2021.682684)
Supplement: Supplementary file 1 [file Table_1.docx]

Table A: Descriptive statistics of the Business Administration and the International Business Administration programs

|  | **Descriptive statistics** | **Business Administration** | | | **International Business Administration** | | | **t-test** |
| --- | --- | --- | --- | --- | --- | --- | --- | --- |
| *Measure* | *Scale Range* | n | *Mean* | *SD* | *n* | *Mean* | *SD* | *p-value* |
| Anxiety (T1) | 1 to 5 | 411 | 1.67 | 0.53 | 310 | 1.74 | 0.68 | 0.09 |
| Depression (T1) | 1 to 5 | 411 | 1.58 | 0.44 | 310 | 1.71 | 0.54 | **0.0005** |
| Denial (T2) | 1 to 5 | 312 | 1.44 | 0.7 | 287 | 1.68 | 0.87 | **0.0003** |
| Behavioral disengagement (T2) | 1 to 5 | 312 | 1.93 | 0.85 | 287 | 1.91 | 0.87 | 0.84 |
| Venting (T2) | 1 to 5 | 312 | 2.25 | 0.81 | 287 | 2.41 | 0.93 | **0.02** |
| Substance Use (T2) | 1 to 5 | 312 | 1.49 | 0.83 | 287 | 1.45 | 0.77 | 0.55 |
| Instrumental Support (T2) | 1 to 5 | 312 | 2.56 | 0.97 | 287 | 2.61 | 1.12 | 0.55 |
| Emotional Support (T2) | 1 to 5 | 312 | 2.99 | 1.02 | 287 | 3.16 | 1.07 | 0.053 |
| Planning (T2) | 1 to 5 | 312 | 3.08 | 0.94 | 287 | 3.28 | 0.96 | **0.01** |
| Positive Framing (T2) | 1 to 5 | 312 | 3.15 | 1.02 | 287 | 3.41 | 0.95 | **0.001** |
| Active Coping (T2) | 1 to 5 | 312 | 3.24 | 0.85 | 287 | 3.3 | 0.82 | 0.39 |
| Loneliness (T2) | 1 to 5 | 295 | 2.39 | 0.71 | 284 | 2.65 | 0.74 | **0.00002** |
| Anxiety (T3) | 1 to 5 | 233 | 1.66 | 0.59 | 192 | 1.8 | 0.77 | **0.03** |
| Depression (T3) | 1 to 5 | 234 | 1.55 | 0.49 | 192 | 1.66 | 0.58 | **0.03** |
| Procrastination (T3) | 1 to 5 | 238 | 2.27 | 0.73 | 193 | 2.6 | 0.75 | **0.000005** |
| ECTs (T4) | 1 to 5 | 443 | 56.73 | 6.09 | 337 | 57.74 | 5.4 | **0.01** |

*Note.* *M* and *SD* are used to represent mean and standard deviation, respectively. In bold, the significant differences.
