# Supplementary material for: Depression and Anxiety in Times of COVID-19: How Coping Strategies and Loneliness Relate to Mental Health Outcomes and Academic Performance
Source: Front Psychol. 2021 Oct 25;12:682684. doi: 10.3389/fpsyg.2021.682684 (PMC8572913; doi:10.3389/fpsyg.2021.682684)
Supplement: Supplementary file 2 [file Table_2.doc]

Table B 
 
Means, standard deviations, and correlations
 
Variable	M	SD	1	2	3	4	5	6	7	8	9	10	11	12	13	14	15	16	17	18	19	
																						
 	 	 	 	 	 	 	 	 	 	 	 	 	 	 	 	 	 	 	 	 	 	
 1. Depression_T1	1.64	0.49	 	 	 	 	 	 	 	 	 	 	 	 	 	 	 	 	 	 	 	
 	 	 	 	 	 	 	 	 	 	 	 	 	 	 	 	 	 	 	 	 	 	
 	 	 	 	 	 	 	 	 	 	 	 	 	 	 	 	 	 	 	 	 	 	
 2. Anxiety T1	1.70	0.60	.75**	 	 	 	 	 	 	 	 	 	 	 	 	 	 	 	 	 	 	
 	 	 	 	 	 	 	 	 	 	 	 	 	 	 	 	 	 	 	 	 	 	
 3. Self-Distraction T2	3.37	0.84	.14**	.19**	 	 	 	 	 	 	 	 	 	 	 	 	 	 	 	 	 	
 	 	 	 	 	 	 	 	 	 	 	 	 	 	 	 	 	 	 	 	 	 	
 4. Active coping T2	3.28	0.84	-.07	-.05	.32**	 	 	 	 	 	 	 	 	 	 	 	 	 	 	 	 	
 	 	 	 	 	 	 	 	 	 	 	 	 	 	 	 	 	 	 	 	 	 	
 5. Denial T2	1.55	0.80	.17**	.15**	.14**	.03	 	 	 	 	 	 	 	 	 	 	 	 	 	 	 	
 	 	 	 	 	 	 	 	 	 	 	 	 	 	 	 	 	 	 	 	 	 	
 6. Substance use T2	1.47	0.80	.17**	.10*	.02	-.04	.28**	 	 	 	 	 	 	 	 	 	 	 	 	 	 	
 	 	 	 	 	 	 	 	 	 	 	 	 	 	 	 	 	 	 	 	 	 	
 7. Emotional Support T2	3.08	1.05	.02	.07	.27**	.28**	.19**	.10*	 	 	 	 	 	 	 	 	 	 	 	 	 	
 	 	 	 	 	 	 	 	 	 	 	 	 	 	 	 	 	 	 	 	 	 	
 8.  Behavioral disengagement T2	1.92	0.86	.34**	.35**	.12**	-.08*	.40**	.26**	.14**	 	 	 	 	 	 	 	 	 	 	 	 	
 	 	 	 	 	 	 	 	 	 	 	 	 	 	 	 	 	 	 	 	 	 	
 9. Venting T2	2.33	0.87	.26**	.25**	.21**	.12**	.37**	.27**	.37**	.36**	 	 	 	 	 	 	 	 	 	 	 	
 	 	 	 	 	 	 	 	 	 	 	 	 	 	 	 	 	 	 	 	 	 	
10. Instrumental Support T2	2.59	1.04	.06	.13**	.27**	.25**	.22**	.13**	.73**	.20**	.46**	 	 	 	 	 	 	 	 	 	 	
 	 	 	 	 	 	 	 	 	 	 	 	 	 	 	 	 	 	 	 	 	 	
11. Positive Framing T2	3.28	1.00	-.02	.05	.27**	.36**	.06	-.03	.37**	.03	.15**	.27**	 	 	 	 	 	 	 	 	 	
 	 	 	 	 	 	 	 	 	 	 	 	 	 	 	 	 	 	 	 	 	 	
12. Planning T2	3.18	0.96	-.04	-.01	.27**	.48**	.13**	-.00	.43**	.02	.31**	.45**	.48**	 	 	 	 	 	 	 	 	
 	 	 	 	 	 	 	 	 	 	 	 	 	 	 	 	 	 	 	 	 	 	
13. Humor T2	2.74	1.08	.12**	.06	.07	.09*	.10*	.13**	.13**	.04	.21**	.08	.23**	.15**	 	 	 	 	 	 	 	
 	 	 	 	 	 	 	 	 	 	 	 	 	 	 	 	 	 	 	 	 	 	
14. Reverse Acceptance T2	1.92	0.76	.16**	.15**	-.06	-.21**	.31**	.19**	-.08*	.25**	.06	-.02	-.26**	-.24**	-.13**	 	 	 	 	 	 	
 	 	 	 	 	 	 	 	 	 	 	 	 	 	 	 	 	 	 	 	 	 	
15. Religion T2	1.66	1.01	.01	.05	.08	.03	.19**	.02	.08*	.08	.08*	.19**	.09*	.18**	-.04	.09*	 	 	 	 	 	
 	 	 	 	 	 	 	 	 	 	 	 	 	 	 	 	 	 	 	 	 	 	
16. Loneliness T2	2.52	0.74	.39**	.35**	.15**	-.17**	.21**	.18**	-.06	.34**	.27**	.03	-.10*	-.06	.00	.33**	.04	 	 	 	 	
 	 	 	 	 	 	 	 	 	 	 	 	 	 	 	 	 	 	 	 	 	 	
17. Procrastination T3	2.42	0.76	.30**	.21**	.10*	-.13**	.11*	.15**	.00	.11*	.12*	-.01	-.02	-.09	.14**	.11*	.04	.19**	 	 	 	
 	 	 	 	 	 	 	 	 	 	 	 	 	 	 	 	 	 	 	 	 	 	
18. Depression T3	1.60	0.54	.61**	.57**	.20**	-.08	.20**	.20**	.05	.30**	.28**	.04	-.01	-.03	.12*	.19**	.14**	.44**	.37**	 	 	
 	 	 	 	 	 	 	 	 	 	 	 	 	 	 	 	 	 	 	 	 	 	
19. Anxiety T3	1.73	0.68	.58**	.67**	.22**	-.03	.23**	.15**	.10	.31**	.29**	.13*	.07	.06	.08	.21**	.14**	.43**	.30**	.79**	 	
 	 	 	 	 	 	 	 	 	 	 	 	 	 	 	 	 	 	 	 	 	 	
20. Academic Performance T4	57.17	5.82	-.11**	-.02	.05	.07	.03	-.04	.08*	-.01	.02	.04	.07	.06	.05	-.09*	-.05	-.09*	-.12*	-.09	-.00	
 	 	 	 	 	 	 	 	 	 	 	 	 	 	 	 	 	 	 	 	 	 	

Note. M and SD are used to represent mean and standard deviation, respectively. * indicates p < .05. ** indicates p < .01.
